# AutoLoc: Autonomous Sensor Location Configuration *via* Cross Modal Sensing

**DOI:** 10.3389/fdata.2022.835949

**Published:** 2022-03-28

**Authors:** Shubham Rohal, Yue Zhang, Carlos Ruiz, Shijia Pan

**Affiliations:** ^1^Computer Science and Engineering, University of California, Merced, Merced, CA, United States; ^2^Aifi Inc., Santa Clara, CA, United States

**Keywords:** vibration based human sensing, autonomous sensor configuration, cross modal sensing, sensor location estimation, multilateration

## Abstract

Internet-of-Things (IoT) systems have become pervasive for smart homes. In recent years, many of these IoT sensing systems are developed to enable in-home long-term monitoring applications, such as personalized services in smart homes, elderly/patient monitoring, etc. However, these systems often require complicated and expensive installation processes, which are some of the main concerns affecting users' adoption of smart home systems. In this work, we focus on floor vibration-based occupant monitoring systems, which enables non-intrusive in-home continuous occupant monitoring, such as patient step tracking and gait analysis. However, to enable these applications, the system would require known locations of vibration sensors placed in the environment. Current practice relies on manually input of location, which makes the installation labor-intensive, time consuming, and expensive. On the other hand, without known location of vibration sensors, the output of the system does not have intuitive physical meaning and is incomprehensive to users, which limits the systems' usability. We present *AutoLoc*, a scheme to estimate the location of the vibration sensors in a two-dimensional space in the view of a nearby camera, which has spatial physical meaning. *AutoLoc* utilizes occupants' walking events captured by both vibration sensors and the co-located camera to estimate the vibration sensors' location in the camera view. First, *AutoLoc* detects and localizes the occupant's footsteps in the vision data. Then, it associates the time and location of the event to the floor vibration data. Next, the extracted vibration data of the given event from multiple vibration sensors are used to estimate the sensors' locations in the camera view coordinates. We conducted real-world experiments and achieved up to 0.07 meters localization accuracy.

## 1. Introduction

Over years, IoT systems became more pervasive to enable human-centric smart building applications, such as patient care, elderly care, and in-home occupant monitoring (Saraubon et al., 2018; Pandey and Litoriya, 2019). These systems infer occupant information, such as identity, location, gait, etc., from ambient non-intrusive sensors, such as WiFi, vibration, powerline (Xu et al., 2013; Mirshekari et al., 2018; Zhou et al., 2019). To enable such functionalities, the systems often require the vibration sensor locations in the room coordinates (Khan et al., 2009; Xiao et al., 2009), which is currently input manually by experts. This results in the complicated installation process and increasing the labor cost, which makes the system less desirable to end-users (Balta-Ozkan et al., 2013). As a result, plug-and-play sensing systems that can **autonomously configure the vibration sensor locations** are required to save users time and reduce dependency on outside experts. This will eventually reduce the deployment cost and increase the scalability of the systems for users without IoT domain knowledge such as caregiver of elderly.

Efforts have been done on sensors' location arrangement estimation (Sun et al., 2011; Kuang et al., 2013; Kamminga et al., 2016) or virtual mapping (Purohit et al., 2013b). However, they can only achieve relative sensor positions estimation, which lacks of the association to the real-world coordinates, e.g., room coordinates. Without an intuitive association to the real-world environment, the system outputs are often not explainable to the end users.

Sensor fusion-based approaches are then explored, leveraging the advantage of the camera as a “helper” modality and inferring the information of other “helpee” modalities. They utilize the cameras in the environment to capture the shared human information—the posture—and establish the association to the other modality, e.g., wearables (Ruiz et al., 2019, 2020) and floor vibration sensing (He et al., 2020). However, the localization resolution of these prior works is limited—either limited to limbs or one spatial dimension. For a generalized sensing system configuration, 2D sensor locations are needed to enable various context-based or collaborative tasks, such as activity recognition and localization.

**Figure 1:**
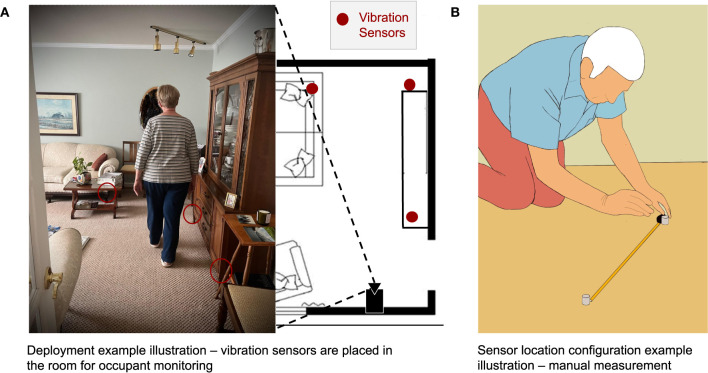
Motivation examples for *AutoLoc*. **(A)** Vibration sensors are deployed in the living room area for application of elderly in-home monitoring. A co-located camera can capture the room view. *AutoLoc* estimates these vibration sensors' location in the room coordinates *via* camera data with or without a line-of-sight to the vibration sensors. **(B)** Manual measurement of these vibration sensor locations is costly and could significantly impact the usability of the system.

In this work, we target the emerging indoor human sensing modality of floor vibration, which provides non-intrusive in-home continuous monitoring, such as patient tracking, gait analysis, behavior profiling, etc. As illustrated in the motivation example in Figure 1, vibration sensing devices are placed in the target sensing area to capture occupant induced floor vibration for human information inference. These sensing devices, similar to Amazon Echo, are semi-mobile and can be easily deployed by placing them on the floor. However, for applications such as patient tracking and gait analysis, they use vibration sensors as anchor devices to infer excitation locations, which requires the prior knowledge of the sensors' locations (Mirshekari et al., 2018; Fagert et al., 2019). Acquiring this information could be troublesome for users, especially those without domain knowledge on how does the system function. Furthermore, even we were able to acquire this prior knowledge for the initial deployment *via* manual measurements, the sensor locations may be updated in a human-in-the-loop manner to optimize system performance (Yu et al., 2021), which would require another round of manual measurements. This would significantly reduce the usability of the system and autonomous sensor location configuration is desirable.

To achieve autonomous vibration sensor location configuration, we present *AutoLoc*, a scheme that repurposes data from vibration sensors and a co-located camera to localize the deployed vibration sensors in a 2-D space. *AutoLoc* does not require the prior knowledge of the camera location, nor line-of-sight (LoS) between the camera and the target vibration sensors. *AutoLoc* achieves this by utilizing the occupant footstep information captured by both the camera and the vibration sensors in their common sensing area. Whenever a person walks through the common sensing area, the camera can detect the occupant's footsteps using pose estimation and localize these footsteps in the target 2-D area (e.g., the living room). The footstep-induced vibration signal propagates through the floor and is captured by multiple vibration sensors in the common sensing area. *AutoLoc* associates the footstep's information—(1) the footstep's location in the camera view and (2) the footstep's relative location to a pair of vibration sensors—between two modalities. To leverage existing cameras people may have in the environment as well as their limited computational power, e.g., smartphone, smart TV, *AutoLoc* adopts light weighted pose estimation model to acquire footstep location in the camera view. To reflect such constraint, we conduct the system evaluation with BlazePose, a lightweight pose estimation model that can run on devices like a smartphone (Bazarevsky et al., 2020). Our system models sensor localization problem as an **inverse** of prior work on multitateration-based footstep localization (Mirshekari et al., 2018). *AutoLoc* estimates 2-D room coordinates of each vibration sensor pair by solving the multilateration equations (Mirshekari et al., 2018) with the footstep locations as known variables and sensor locations as unknown variables.

Challenges for *AutoLoc* include (1) how to accurate footstep event detection and localization *via* video signal with noisy posture estimation? (2) how to solve multilateration equations with unknown vibration propagation velocity? and (3) how to select a subset of footstep events from accumulating sensor data to achieve accurate vibration sensor localization? *AutoLoc* tackles these challenges by combining physical and data-driven knowledge (details in section 3) and achieves an up to 0.07 meters localization accuracy for co-located vibration sensors in real-world experiments, which is an up to 4× improvement compared to baselines. We summarize the contribution of this work as follows.

- We present *AutoLoc*, a cross-modal scheme for autonomous sensing device 2-D location configuration.
- We use physical knowledge on surface wave propagation properties to determine equation constraints for accurate multilateration solutions.
- We design a spatial-aware algorithm that takes physical location information of data inputs into account to improve the sensor location estimation accuracy
- We conduct real-world experiments to evaluate the scheme.

The rest of the paper is organized as follows. First, we discuss the scope of this work compared to the prior works in section 2. Next, we introduce the detailed system design in section 3. Then, we provide system evaluation with real-world deployment comparing to multiple baselines in section 4. Finally, we discuss the potential future direction in section 5 and conclude this work in section 6.

## 2. Related Work

We summarize relevant prior work in this section and compare *AutoLoc* to them.

### 2.1. Vibration-Based Human Sensing

Physical vibration signals induced by people in the buildings are used to indirectly infer human information for both physical and physiology information, include and not limited to identity (Pan et al., 2017), location (Mirshekari et al., 2018; Drira et al., 2021), activity (Hu et al., 2020; Sun et al., 2020), heart beat (Jia et al., 2016), and gait (Fagert et al., 2019). The intuition is that people induce physical vibrations all the time, such as stepping on the floor, heart-pounding in the chest, etc. Vibration sensors placed on the ambient surfaces can capture these vibrations propagating through the surface and infer the source of the signal. These prior works demonstrate the feasibility and potential of the physical vibration-based sensing system for various human-centric applications, which validates the motivation of this work.

System often require sensors to have overlapping sensing area to enable applications such as step-level localization (Mirshekari et al., 2018), gait analysis (Fagert et al., 2020), and activity recognition (Hu et al., 2020; Sun et al., 2020). On the other hands, for applications such as localization and gait analysis, sensor devices' locations in the room coordinates are also needed. Therefore, autonomous sensor location configuration is important for these vibration-based human sensing applications.

### 2.2. Device Localization

Localizing devices have been explored widely for robotics and mobile-based approaches. Various approaches have been explored over many sensing modalities. Landmark based approaches were adopted as data-driven approaches over visual landmarks (Se et al., 2002), infrared light landmark (Lee and Song, 2007), RF landmark (Purohit et al., 2013a), etc. On the other hand, multilateration is a commonly used approach as physics based approaches. It has been applied on acoustic- (Höflinger et al., 2014), WiFi- (Arthi et al., 2020), UWB- (Onalaja et al., 2014), BLE- (Shchekotov and Shilov, 2018) based systems. These devices and systems are mostly equipped with transceivers for ranging purpose. Acoustic-based devices relative physical arrangement detection problem is also being explored, where the sources of the signal and the devices are localized simultaneously (Sun et al., 2011; Kuang et al., 2013; Kamminga et al., 2016). However, they were able to achieve the relative arrangement of the device rather than the absolute physical locations. As a result, their target application/usage is also different from what we target in this work. In addition, the signal and sensing modality targeted in this paper—building vibration based occupant monitoring—faces more challenges such as high decay rate, high distortion, dispersion and ambient noise compared to prior work of acoustic-based sensing.

### 2.3. Cross-Modal Autonomous System Configuration

Multiple co-located sensing modalities are used to enable automation of system configuration. These modalities are associated over the shared context (both spatial and temporal) in the physical world that can be captured by different types of sensors (Han et al., 2018; Pan et al., 2018; Yu et al., 2019; He et al., 2020). Motion has been used as the shared context between IMUs on wearables and camera view to enable auto-pairing for IoT devices (Pan et al., 2018). Event timing is another type of shared context that is used to generate encryption keys for secured pairing (Han et al., 2018). These systems relies on a **direct measurable** context for both sensing modalities. However, when there is no directly measurable shared context, the **indirect inference** introduce more challenges, such as vibration-based sensing modalities. Footstep location has been used as a shared context to associate the vibration devices absolute locations (He et al., 2020). However, it is only been explored in a 1-D scenario, which is not sufficient for some of the applications such as localization and activity recognition. In this work, we focus on the 2-D solution of the vibration sensor localization *via* camera captured ambient occupant context.

## 3. System Design

**Figure 2:**
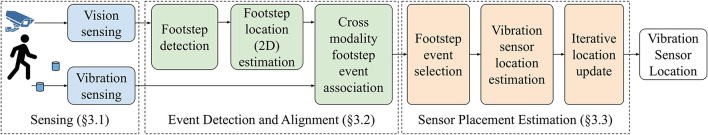
System overview.

The system consists of mainly three modules, the sensing module (section 3.1), the event detection and alignment module (section 3.2), and sensor placement estimation module (section 3.3), as shown in Figure 2. First, the Sensing module takes both structural vibration and vision data as inputs. Next, the Event Detection and Alignment module detects the footstep using a light-weighted posture estimation on vision data, and uses the detected heel keypoints to acquire 2-D coordinates of the footstep in the common sensing area. *AutoLoc* segments the structural vibration signal using the timestamp of this detected footstep and assigns this footstep's 2D coordinates to the segment of signal. However, due to the noisy output of the light-weighted posture estimation model, the raw signal association may be erroneous, which leads to high localization error in the final estimation. We leverages physical knowledge on the human-induced vibration signal quality to select the subset of signals and ensure high estimation accuracy. Thus, Sensor Placement Estimation module first selects the footstep event with a signal-to-noise ratio (SNR) higher than a threshold and over different areas. *AutoLoc* then estimates sensors' locations using the selected known footstep locations *via* multilateration. To further reduce the estimation error, *AutoLoc* updates the sensors' locations iteratively when more footstep events are acquired.

### 3.1. Sensing Module: Application and Assumptions

*AutoLoc* takes both vision and floor vibration sensing data as inputs and they are synchronized to second-level. We consider the floor vibration sensing systems are deployed in the home environment—the sensing devices are placed on the floor by the wall or under the furniture—as the example shown in Figure 1. When occupants walk in their home, their footsteps induce floor vibrations, which can be captured by the vibration sensors nearby.

We assume two scenarios for the camera setup. (1) Some area in the home is already covered by a camera, e.g., smart TV with a built-in camera (Samsung Inc., 2017) in the living room or surveillance camera in the doorway, and can be re-purposed for autonomous configuration purposes. (2) A device with camera, e.g., a smartphone, is temporarily set up for a short period of time for autonomous configuration purposes. The camera captures sensing areas overlapping deployed vibration sensors' sensing areas, which we refer to as their common sensing area.

### 3.2. Footstep Event Detection and Alignment

*AutoLoc* takes synchronized vision and vibration signals as inputs and outputs the footstep-induced vibration signals and their locations in *x* and *y* coordinates. First, *AutoLoc* extracts the occupant's heel keypoints on vision data *via* pose estimation (section 3.2.1). Then, these heel keypoints are used to detect the occupant's footstep events (section 3.2.2). Next, the heel keypoints extracted by the pose estimation are used to acquire footstep event's 2D location (section 3.2.3). Finally, *AutoLoc* extracts the vibration signal induced by the detected footstep event using event timestamp and associates it with the estimated location (section 3.2.4).

#### 3.2.1. Heel Keypoint Tracking

**Figure 3:**
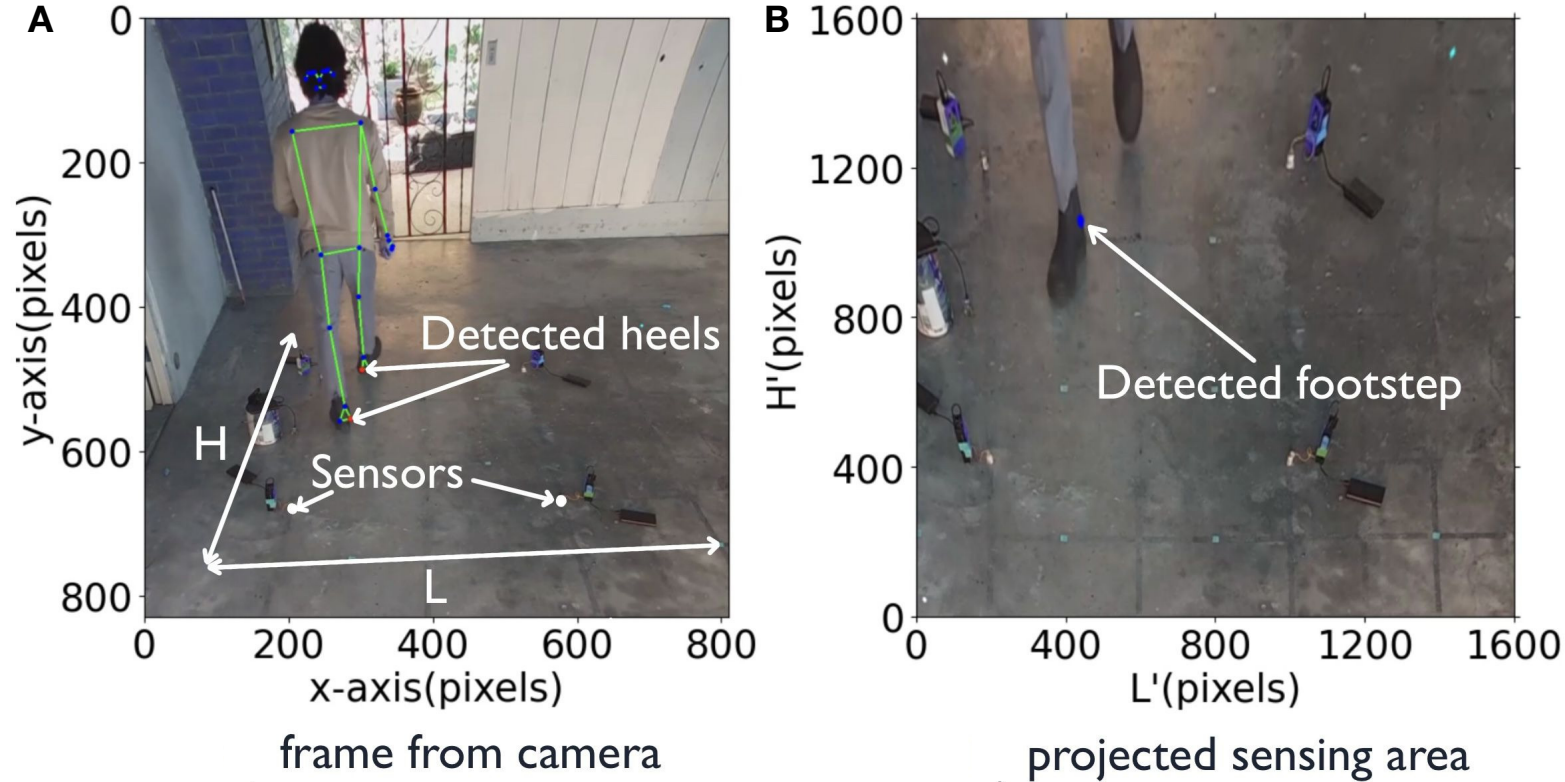
Pedestrian posture estimation for footstep event detection **(A)** BlazePose outputs keypoints of human posture marked in blue dots. The red dot marks the detected heel keypoints. AutoLoc tracks the heel keypoints for footstep detection. **(B)** Sensing area H' × L' in the floor coordinate projected from H × L in the camera view **(A)**.

*AutoLoc* uses pose estimation model to detect the posture of occupants in the sensing area. To make sure the pose estimation is not too demanding computationally we used BlazePose (Bazarevsky et al., 2020). BlazePose is a lightweight convolutional neural network for pose estimation that can run devices with less computational power and user already owns like smartphone (Bazarevsky et al., 2020). The model takes raw frames from video data as the input and outputs the coordinates of 33 keypoints of the human posture in that frame. Figure 3A demonstrates an example camera view and the pedestrian posture captured with keypoints marked in red dots. Among these 33 keypoints we track the position of two heel keypoints since our goal is to extract the footstep timing of the heel strikes, which induce floor vibration.

#### 3.2.2. Vision-Based Footstep Event Detection

**Figure 4:**
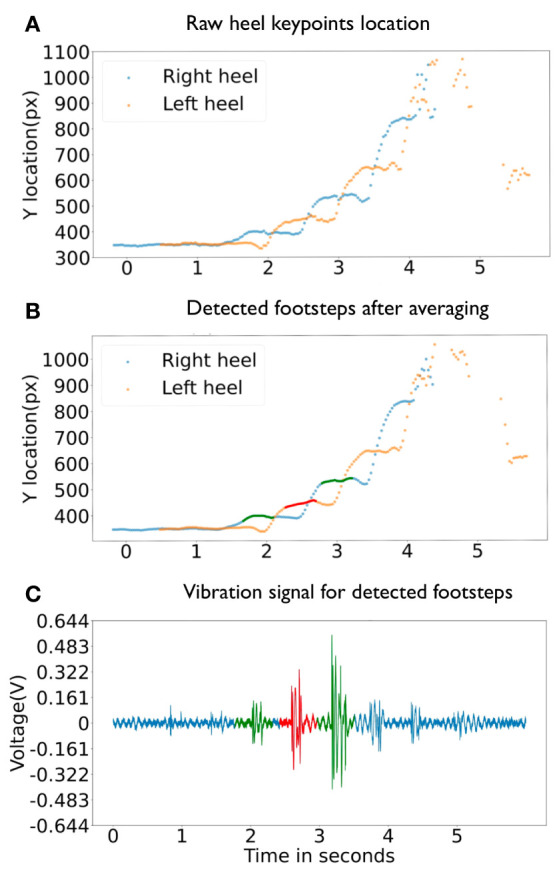
Cross-modal footstep event signal association example. **(A)** shows heel keypoints location detected in video frames before smoothing. **(B)** shows heel keypoints location detected in video frames after smoothing. The red and greed dots mark detected footstep event. **(C)** shows the floor vibration signal in blue lines and extracted footstep event signal segments detection by the cross-modal context in red and green segments.

We define a **footstep event** from heel strike to pre swing phase in the gait cycle (Whittle, 2014). The intuition of the event detection is that during this phase, the corresponding heel does not move. However, due to the noisy output of the light-weighted posture estimation module, the estimated location of heel keypoint may fluctuate by a few pixels. To tackle this problem, we apply the moving average of a window size *swVision* to smooth the heel keypoints position. Figures 4A,B show detected heel keypoints before and after applying the sliding window. The smoothed heel keypoint position at time *t* is denoted as *heelP*_*t*_.

Algorithm 1 depicts details of the event detection. *AutoLoc* takes the heel keypoint location at time *t* – *heelP*_*t*_ as the input. If the distance between *heelP*_*t*_ and *heelP*_*t* − 1_ is less than *motionTh*, the system considers that the foot is not moving and increments the counter *stableFrame*. If the distance between *heelP*_*t*_ and *heelP*_*t*−1_ is larger than *motionTh*, the system considers it as the end of a potential footstep and checks *stableFrame* value. If the *stableFrame* value is larger than the *frameTh* value, the system assigns footstep event time *eTime* as *t* − *stableFrame*/2. After detecting the footstep event at *eTime*, *AutoLoc* calculates *eLoc*, the pixel location of the footstep event in the camera view, as the mean of the heel keypoint locations from time *t* − *stableFrame* to *t* − 1.

**Algorithm 1:**
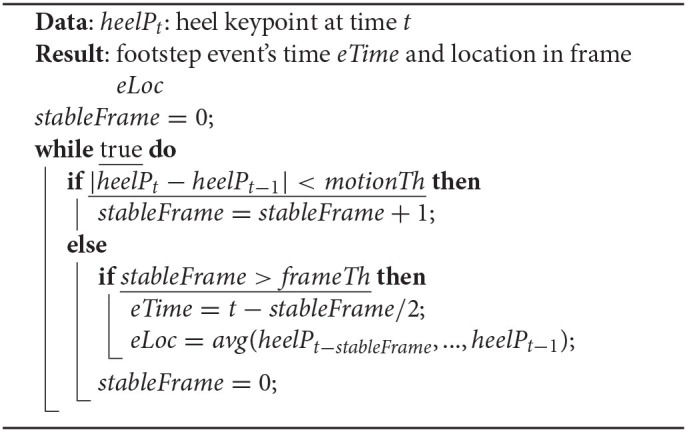
Vision-based footstep event detection.

#### 3.2.3. Vision-Based Footstep Localization

We predefined floor coordinates in the camera view to associate footstep's pixel location to it. Figure 3 shows an example of (a) the floor coordinates *H* × *L* in the camera view and (b) the projected floor coordinate *H*′ × *L*′. This projection requires a 3 × 3 transformation matrix, which can be computed using the coordinated four points of the sensing area in the camera view and the coordinates of the transformed image (Forsyth and Ponce, 2011).


(1) $$\begin{array}{l} \left[\begin{array}{c} x^{\prime }\\ y^{\prime }\\ 1 \end{array}\right]=\left[\begin{array}{ccc} a_{1} & a_{2} & b_{1}\\ a_{3} & a_{4} & b_{2}\\ c_{1} & c_{2} & 1 \end{array}\right]\left[\begin{array}{c} x\\ y\\ 1 \end{array}\right]\\ C^{\prime }=MC \end{array}$$

Here, *x, y* in *C* represents the coordinates of one point of sensing area in the camera view, i.e., *eLoc*, and 1 is the scaling factor, while *x*′, *y*′ in *C*′ represents the new coordinates of the corresponding point in the transformed image, i.e., *fLoc*,. *M* is the transformation matrix, in which the 2 × 2 upper left matrix $\left[\begin{array}{cc} a_{1} & a_{2}\\ a_{3} & a_{4} \end{array}\right]$ defines scaling and rotation transformation. The two elements of right column $\left[\begin{array}{c} b_{1}\\ b_{2} \end{array}\right]$defines translation vector and in last row[*c*_1_, *c*_2_] defines projection vector (Forsyth and Ponce, 2011). Given *C* and *C*′, the transform matrix *M* is estimated by solving the overdetermined linear system. Then the transformed heel keypoint $\left(x_{h}^{\prime },y_{h}^{\prime }\right)$ can be further calculated by Equation (1) given *M* and *x*_*h*_, *y*_*h*_. $\left(x_{h}^{\prime },y_{h}^{\prime }\right)$ is under the new coordinate (*H*′ × *L*′), which is the physical area location. Figure 3B shows an example of the sensing area view transformed from the original camera view in (a).

In addition, the heel keypoints detected by the pose estimation algorithm are not the precise contact points between the foot and the floor due to the shoe-induced offset and camera view perspective. As a result, *AutoLoc* empirically adds an offset (x^*^, y^*^) to the estimated heel location $\left(x_{h}^{\prime },y_{h}^{\prime }\right)$ as the footstep location.

#### 3.2.4. Footstep-Induced Vibration Signal Location Association

Once we acquire the time and location of the footstep events from the camera data, we further derive the vibration sensors' locations. Because these two sensing modalities capture the same footstep event at two different gait cycles—the camera captures the footstep at the terminal stance phase, while the vibration sensor captures the footstep at the initial contact phase—*AutoLoc* have to compensate this cycle difference to ensure accurate association. For the footstep event detected at the time *eTime* by the camera, the foot-floor initial contact would occur before the heel motion is detected as stable since there is a detection delay. As a result, *AutoLoc* segments the vibration signal between *eTime* − *eDelay* and *eTime* − *eDelay* + *eDuration* as the footstep event. The configuration of these values will be detailed in section 4. Figure 4 depicts an example of detected footsteps and their association between (a) the camera data and (b) the vibration signal.

### 3.3. Vibration Sensor Location Estimation

When occupants pass by the common sensing area *H* × *L* multiple times, their detected footstep events accumulate. With the footstep event signals extracted and their locations estimated, *AutoLoc* further calculates the vibration sensors' location. To do so, *AutoLoc* solves the multilateration equations with locations of footsteps as the known variables and sensor locations as unknown variables. In this way, *AutoLoc* simultaneously localizes the pair of vibration sensors that capture the footstep event.

#### 3.3.1. Inverted Problem for Step-Level Localization

We model the vibration sensor localization problem in the same form as the multilateration-based footstep localization, but with inverted known and unknown variables.

In the footstep localization problem, locations of the deployed floor vibration sensors are known. Based on the vibration sensors outputs, the system measures Time Difference of Arrival (TDoA) between sensor pairs and conducts TDoA-based multilateration by solving the set of equations for footstep locations (Mirshekari et al., 2018).

In our problem setting, footstep locations can be estimated using the vision data as discussed in section 3.2.3. Therefore, we consider them as the known variables. On the other hand, the locations of the pair of sensors *sLoc*_1_ and *sLoc*_2_ are unknown variables. When a footstep event occurs at location *fLoc* at time *t*_0_, its vibration signal propagates through the floor surface and reaches the sensor located at *sLoc* at time *t*_*s*_. This physical process can be described as


(2) $$\begin{array}{l} \left(t_{s}-t_{0}\right)v=||sLoc-fLoc|{|}_{2} \end{array}$$

where *v* is the wave propagation velocity. Due to camera's low sampling rate and noisy vision-based footstep detection, the measurement of *t*_0_ is not accurate enough for the propagation time measurement.

To counter this problem, *AutoLoc* utilizes pairwise sensors monitoring the same area and localize them *via* Time Difference of Arrival (TDoA) based multilateration. We consider the pairwise sensors at the positions (*sLoc*_1_) and (*sLoc*_2_). They receive the signal from same footstep at the location *fLoc* at time *t*_1_ and *t*_2_ respectively. Based on Equation (2), *AutoLoc* acquires the TDoA of these pairwise sensors as follows.


(3) $$\begin{array}{l} \left(t_{1}-t_{2}\right)\times v=TDoA\times v=||sLoc_{1}-fLoc|{|}_{2}-||sLoc_{2}-fLoc|{|}_{2} \end{array}$$

The TDoA between signals captured by these pairwise sensors can be calculated as the time domain shift, which we will discuss in detail in section 3.3.2.

#### 3.3.2. Time Difference of Arrival (TDoA) Measurements

Acquiring accurate TDoA measurements from vibration signals propagating over floor is challenging because of the dispersion effects (Mirshekari et al., 2018). The dispersion effects result in different propagation velocities over different frequency band, which directly impact the signal waveform that are used for calculating their time domain shift. Wavelet decomposition and filtering has been proven to be efficient for reducing the dispersion effects (Mirshekari et al., 2018). As a result, *AutoLoc* first applies the wavelet filter of frequency band *filtBand* to the signal. Then, *AutoLoc* measures the time domain shift by subtracting the time of the first peak detected in the filtered signal. The first peak is selected as the peak within the footstep event signal segment with a height no less than 1/*K* of the highest value of this signal segment.

#### 3.3.3. Solving Non-linear Least Square Problems With Physical Constraints

Note that for the 2-D localization problem, the unknown variables in Equation (3) are *v*, and 2-D coordinates of *sLoc*_1_ and *sLoc*_2_. However, an impractical solution where *v* = 0 and *sLoc*_1_ = *sLoc*_2_ with any arbitrary values would satisfy these equations. To avoid this solution, *AutoLoc* applies two **physical constraints**: (1) the range of the wave propagation velocity based on the prior knowledge on surface waves; and (2) the range of the possible sensor location based on the vibration sensing range limitations.

For a given wave propagation velocity *v*, *AutoLoc* constructs a non-linear least square problem with *m* footstep induced signal at location *fLoc*_*i*_, *i* = 1...*m* based on Equation (3).


(4) $$\begin{array}{l} f_{i}{\left(x\right)}^{2}=\left(||sLoc_{1}-{fLoc}_{i}|{|}_{2}-||sLoc_{2}-{fLoc}_{i}|{|}_{2}\right)-TDoA_{i}\times v \end{array}$$

$$\begin{array}{l} minimize\;f\left(x\right)=\overset{m}{\underset{n=1}{\sum }}f_{i}{\left(x\right)}^{2} \end{array}$$

x fonts inconsistency where *x* is a vector contains the unknown coordinates of *sLoc*_1_ and *sLoc*_2_. We used Trust-region Reflective (TRF) Optimization (Branch et al., 1999) algorithm to solve the non-linear least squares problem, which is empirically used due to its robustness and ability to solve ill-conditioned problems (Yuan, 2000). TRF is a bound constrained non-linear minimization algorithm, which is based on Newton's Method of root finding. It is an iterative procedure, which represent objective function as quadratic model implied by Taylor series expansion at the current point. At the *kth* iteration, the model computes


(5) $$\begin{array}{ll} minimize & \;\psi_{k}\left(s\right)=g_{k}^{T}s+\frac{1}{2}s^{T}\left(H_{k}+C_{k}\right)s,\\ \;||D_{k}s||\leq \Delta_{k} \end{array}$$

where *g*_*k*_ = ∇*f*(*x*_*k*_) is the gradient and $H_{k}=\nabla^{2}f\left(x_{k}\right)$ is Hessian matrix at the current iteration (Yuan, 2000). *D*_*k*_, *C*_*k*_ are affine scaling matrices and Δ_*k*_ is the trust region bound on the step. By solving Equation (5), we compute a step *s*_*k*_ and value of *x* is updated along with the trust region Δ_*k*_. The affine matrix make sure the step is strictly feasible and pointing inside the bounds. The iteration stops when ||*g*_*k*_||_2_ < ϵ. On the other hand, to select the proper velocity value *v*, *AutoLoc* conducts a grid search on *v* in the range of the surface wave propagation velocities find out the number and cite and outputs the results with the minimized cost function of *f*(*x*) (Yuan, 2000).

#### 3.3.4. Spatial Characterization and Data Selection

*AutoLoc* localizes a pair of sensors at a time by solving the non-linear least square problem discussed in section 3.3.1. The physics guided model—in our case, the multilateration model—is often more sensitive to noise than the data-driven model. Therefore, a data-driven selection procedure is essential to ensure the accuracy of the model. *AutoLoc* tackles this challenge from two aspects.

First of all, the error and noise of the TDoA measurements would impact the sensor localization accuracy. Intuitively, when the vibration signal's Signal-to-Noise Ratio (SNR) is low, the signal is impacted by noise more and has a higher chance to result in errors of the TDoA measurements. To improve the TDoA measurement over the pair of sensors, *AutoLoc* selects footstep events with high fidelity for the task—high SNR for the target pair of sensors—for sensor localization. For a detected event, *AutoLoc* measures the SNR of the event signals captured by both sensors in the target pair. If both signals' SNR is higher than the threshold *sFidelity*, *AutoLoc* consider its spatial characteristics is valid for sensor localization.

Secondly, other than the individual footstep event spatial characteristics, the spatial characteristics of the group of events used in Equation (4) is also important. If the TDoA estimations are all similar, the solution can be impacted by the noise significantly. *AutoLoc* ensures to capture footstep events with high spatial variance to ensure the accuracy of the sensor location estimation by selecting events from different subareas to solve the non-linear least square problem.

## 4. Experiments and Evaluation

We conduct real-world experiments to evaluate *AutoLoc*. In this section, we first introduce the hardware and deployment followed by the data collection procedure in section 4.1. Then, we listed detailed algorithm implementation settings and baselines in section 4.2. Finally, we present results and provide analysis to demonstrate the accuracy and robustness of *AutoLoc* in section 4.3.

### 4.1. Hardware and Data Collection

**Figure 5:**
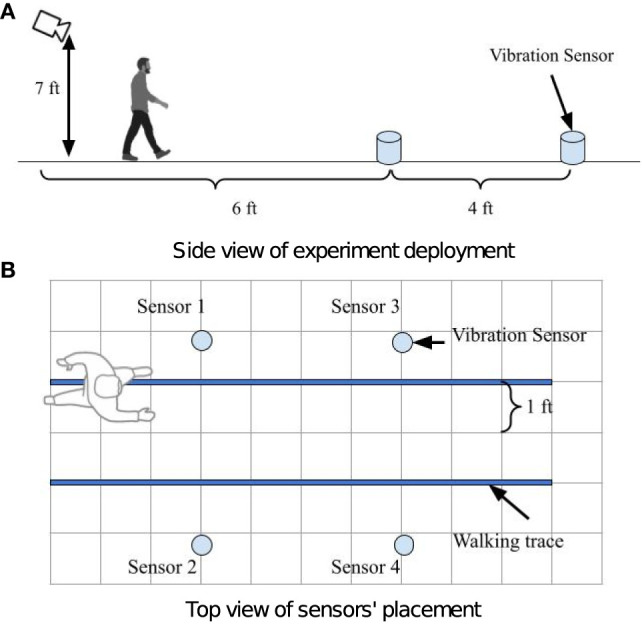
Experiment deployment illustration. A camera is deployed 6 ft away from the sensing area as shown in **(A)**. Four vibration sensors are deployed in the sensing area 4 ft apart as shown in **(B)**. The blue lines in **(B)** depict walking traces of the pedestrian.

Figure 5 demonstrate the setup at the sensing area, where the camera is placed at a height of 7ft from the floor. We consider this setup because it is common in home scenario where an existing surveillance camera or smart TV's top camera is available. These cameras often captures the hallway or living room area where occupants walk by. A commercial camera (Amcrest IP4M-1028EB-28MM) amc (Amcrest Camra Manual, 2019) is used to record the video at resolution of 2, 688 × 1, 520 and 30 frames per second. Four floor vibration sensors are set up on a concrete floor as shown in Figure 5B. Geophone SM-24 (Input/Output Inc, 2006) vibration sensors are used to capture the floor vibrations. The magnitude of footstep-induced vibration signals from the sensors is approximately 10^−3^ to 10^−2^ Volt. In order to ensure the digitized signal resolution for later analysis, we use a low-voltage rail-to-rail OpAmp LMV385 lmv (Operational Amplifier, 2020) to amplify the vibration sensor's signal before it is converted to digital signals. Finally, the amplified signal is digitized by the Analog-to-Digital Converter. Vibration sensors are synchronized to microsecond-level. The four sensors' (Sensor 1–4) locations in the floor coordinates are (0.31, 0.61), (1.52, 0.61), (0.31, 1.83), (1.52, 1.83) in meters, respectively, as shown in Figure 5B. We consider this setup based on prior work's deployment setting on floor vibration based pedestrian localization and gait analysis (Mirshekari et al., 2018; Fagert et al., 2020; Shi et al., 2020). The occupant walks through the target sensing area following the walking traces marked as the solid blue lines. The occupant passes by the common sensing area back and forth 16 times following each path marked by the blue line.

### 4.2. Implementation of *AutoLoc* and Baselines

We implement *AutoLoc* as discussed in section 3 with key parameters setting listed in Table 1. For these experiments, we refer to solving the non-linear least square problem and get a pairwise sensor location with four footstep events as one **estimation**. We consider two subareas as two walking trace paths. For each estimation, *AutoLoc* first randomly selects a trace out of 16 from one subarea. Then *AutoLoc* extracts the detected footfall events that have an SNR higher than the threshold *sFidelity*. If the extracted event number is no less than two, *AutoLoc* outputs the two events with the highest SNR for localization. Otherwise, *AutoLoc* randomly selects another trace from the rest of the traces until at least two events are extracted. Then, *AutoLoc* does the same selection procedure for the other subarea and outputs two events for localization. The selected footsteps are used to construct a non-linear problem as discussed in Equation (4). *AutoLoc* uses the TRF Optimization algorithm to solve this non-linear problem for sensor location estimation with constrained boundaries for four unknown variables.

**Table 1:** *AutoLoc* implementation parameters.

**Parameters**	**Values**	**Description**
*motionTh*	9 pixel	Threshold for heel motion detection
*frameTh*	15 frames	Threshold for footfall event detection
*swVision*	4 frames	Vision smoothing sliding window
(x^*^, y^*^)	(0, 0.05) m	Heel point calibration offset
*eDelay*	0.3 sec	Footfall event detection delay
*eDuration*	0.5 sec	Segment event duration
*filtBand*	7	Wavelet filtering band
*K*	4	First peak detection threshold
*H*′ × *L*′	8*ft* × 8*ft*	Target sensing area

To demonstrate the advantage of the Spatial Characterization and Data Selection, we compare our approach with two baselines.

**Baseline 1: Random Selection**. This baseline keeps on selecting a trace at random from all 32 traces (16 in each subarea) without repeating, until it accumulates four or more footsteps events. Then, the first four events are selected to form a non-linear problem. Then least square method is used to solve non-linear problem for sensor location estimation.

**Baseline 2: SNR-based Selection**. This baseline randomly selects traces from total 32 traces without replacement until it collects at least four detected footfall events with SNR higher than the threshold *sFidelity*. Once four or more footsteps are collected, we select the four events with the highest SNR to conduct sensor localization.

Note that each method (Baseline 1, Baseline 2, and *AutoLoc*) is repeated 1,000 times to ensure the selected trace combinations are not biased.

*AutoLoc* considers the localization fail under the following conditions: (1) when the number of selected footstep events are less than four, (2) when at least one of the sensors (from the target pair) estimated location is on the constrained boundaries of the non-linear least square problem setting. As a result, we use the following metrics to evaluate the system performance: (1) localization error: as the Euclidean distance between the estimated sensor location and the ground truth; (2) localization success rate: as another metric.

### 4.3. Results and Analysis

In this section, we present the results of the vibration sensor localization and analyze them over multiple aspects. We first demonstrate the robustness of the Spatial Characterization and Data Selection scheme (section 3.3.4) by comparing the localization error and success rate to those of the baselines in section 4.3.1. Next, we explored the system parameters (e.g., threshold values) and their impact on the sensor localization accuracy in section 4.3.2. Finally, we demonstrate that with more iterations of location estimation, *AutoLoc* can further refine the final estimation and enhance the accuracy in section 4.3.3.

#### 4.3.1. Data Selection Scheme Comparison

**Figure 6:**
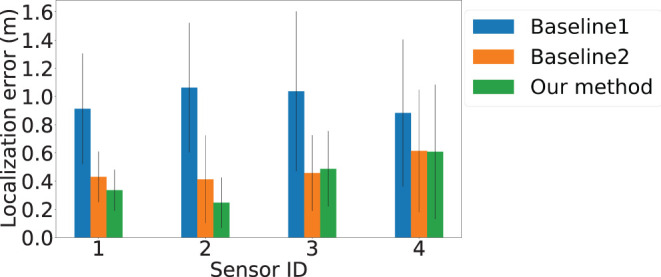
Localization errors of sensors over different locations. The *x*-axis is the Sensor ID. The *y*-axis is the localization errors. Blue, orange, and green bars represent results from Baseline 1, Baseline 2, and our approach *AutoLoc*, respectively. Sensor 3 and 4 demonstrate higher localization error, which could be caused by lower vision-based footfall localization accuracy due to the farther distance between the camera and selected footfall events.

We compare the performance of *AutoLoc* to the two baselines over four sensors' localization accuracy in Figure 6. The *x*-axis is the sensor ID and the *y*-axis is the localization error in meters. The average error using Baseline 1 and 2 are depicted in blue and orange bars with the standard deviation plotted as error bars. The corresponding results of *AutoLoc* is presented in green bars with error bars depicting the standard deviation. We observe that for Baseline 1, where the footfall events are not constrained by SNR, result the highest average localization errors of 0.91, 1.06, 1.03, and 0.88 m for the four sensors, respectively. With the constraint on SNR, Baseline 2 achieves average localization errors of 0.43, 0.41, 0.47, and 0.61 m, respectively. *AutoLoc* applies another constraint on spatial-variance and achieves average localization errors of 0.33, 0.24, 0.49, and 0.61 m, which shows an up to 4× improvement compare to the baselines.

**Figure 7:**
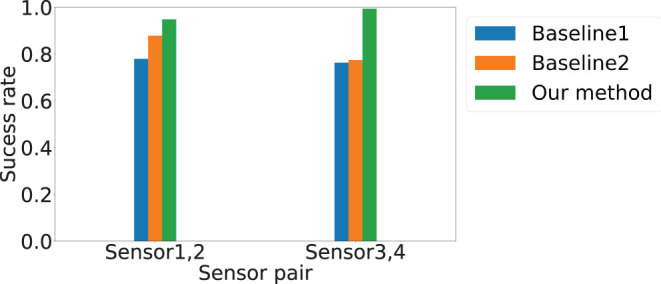
Localization success rate for different sensor pairs. Blue, orange, and green bars represent results from Baseline 1, Baseline 2, and *AutoLoc*, respectively.

Since the system discard the estimation that is exactly on the boundary, we further depict the success rate of estimations in Figure 7. The *x*-axis is the sensor pair, and the *y*-axis is the success rate. We observe that *AutoLoc* not only achieves the highest localization accuracy, it also achieves the highest success rate among the three approaches, indicating the importance of the high quality data selection. For Baseline 1 and 2, the success rate for the pair Sensor 1-2 (78.8 and 87.8) show slightly higher success rate than that of the pair Sensor 3-4 (76.3 and 77.4). Our approach *AutoLoc* achieves 0.95 and 0.99 success rate for the sensor pair Sensor 1-2 and 3-4, respectively.

#### 4.3.2. Impact of Vision Based Detection Thresholds

Since the footfall events' spatial and temporal information relies on vision-based event detection, we investigate the relevant parameters (*motionTh* and *frameTh*) to understand their impacts to *AutoLoc*.

**Figure 8:**
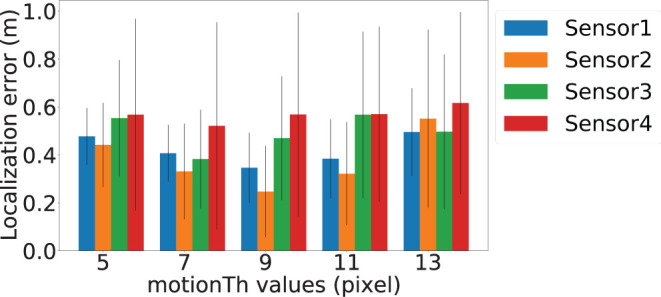
Vision-based footfall event detection parameter *motionTh* and its impact on localization error. Blue, orange, green, and red bars represent Sensor 1, Sensor 2, Sensor 3, and Sensor 4's localization error, respectively. The error bars depict standard deviation values.

**Figure 9:**
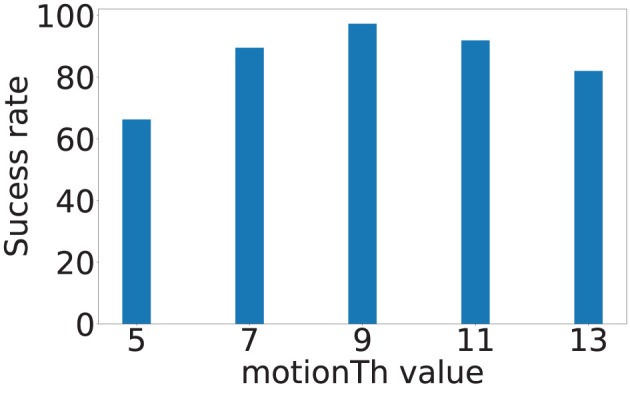
Vision-based footfall event detection parameter *motionTh* and its impact on localization success rate.

*motionTh* determines the system's sensitivity to noise within the posture estimation keypoints. Figure 8 depicts the localization error of the four sensors at different *motionTh* values. The *x*-axis is the *motionTh* value with unit in pixels, and the *y*-axis is the localization errors in meters. We observe that impact of the *motionTh* is more significant for Sensors 1 and 2 compared to Sensors 3 and 4. This could be caused by difference in distance between camera and sensor pair's sensitive area. The localization errors are the lowest when *motionTh* = 9 for Sensor 1 and Sensor 2, which are 0.34 and 0.25 m, respectively. When the *motionTh* increases, the tolerance of motion noise is increased, which lead to a higher false positive rate for footfall event detection. As a result, we observe higher localization errors (0.49, 0.55, 0.49, and 0.61 m, respectively) when *motionTh* = 13. On the other hand, when the *motionTh* decreases, the false negative rate of footfall event detection increase, which reduces the spatial-variance of the events that can be used. This reduction in spatial-variance results a higher localization errors. For example localization error is (0.48, 0.44, 0.55, and 0.57, respectively), when *motionTh* = 5. We also analyze the localization success rate and depict the results in Figure 9. The *x*-axis is the *motionTh* value and the *y*-axis is the success rate. We observe that when *motionTh* = 9 the system achieves the highest success rate of 0.97. When *motionTh* decreases, the false positive detection will lead to more errors, i.e., the estimation falls on constraint bounds.

To understand the effect of *motionTh* on vision-based footstep detection over different distances, we analyze the precision and recall scores of the footstep detection module when footsteps are of different distance to the camera. Considering the center of our target sensing area is 8ft away from the camera, we analyze the results of footsteps that are closer than 8ft away from the camera and those that are farther than 8ft away from the camera.

**Figure 10:**
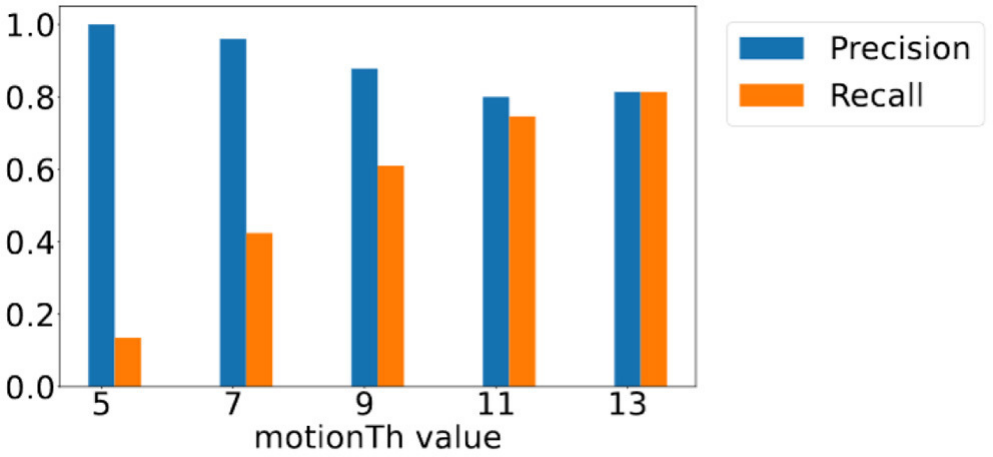
Precision and recall score for footsteps closer than 8 ft away from camera with different *motionTh* values.

Figure 10 shows the precision and recall scores of footsteps that are closer than 8ft away from the camera when different *motionTh* values are adopted. The recall score (orange bars) increases as the value of *motionTh* increases, because less footsteps will be discarded due to the heel keypoint fluctuation from the noisy pose estimation. This decreases the number of footsteps that are not detected (false negative). However, because of this, the number of false positive detection also increases, which causes the decreasing trend in precision score (blue bars) as *motionTh* increases.

**Figure 11:**
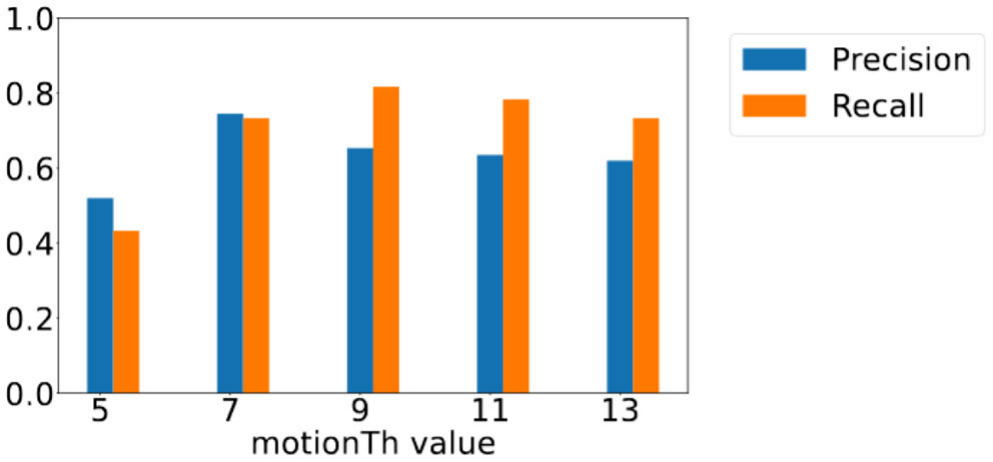
Precision and recall score for footsteps farther than 8 ft away from camera with different *motionTh* values.

Figure 11 shows those of footsteps that are farther than 8ft away from the camera. We observe that the changes of the precision and recall score are less significant compared to those are closer to the camera, and yet they bear the similar overall trends—the precision score shows a decreasing trend for *motionTh* values between 7 and 13 and the recall score shows an increase trend between 5 and 9. Precision score is lower for *motionTh* value 5 in the farther distance area, because the farther distance leads to less pixel changes for the same amount of motion, causing a high false positive when *motionTh* is low, i.e., value 5.

**Figure 12:**
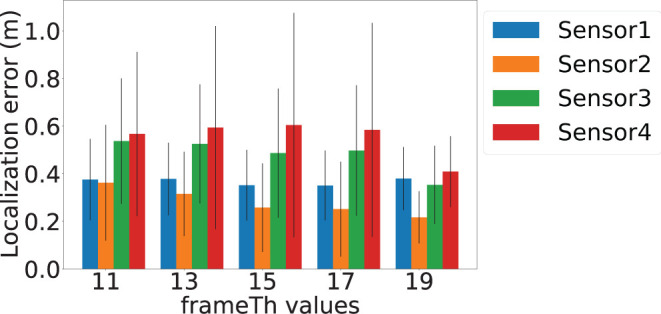
Vision-based footstep event detection parameter *frameTh* and its impact on localization error.

**Figure 13:**
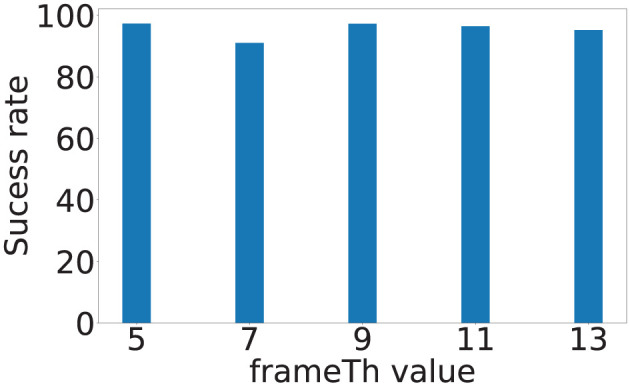
Vision-based footstep event detection parameter *frameTh* and its impact on success rate.

**Figure 14:**
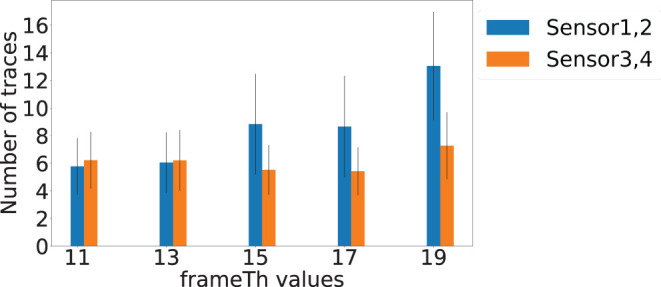
Number of traces sampled when *frameTh* vary. The *x*-axis is the *frameTh* value and the *y*-axis is the number of traces sampled till four footfall events that met constraints being extracted.

The *frameTh* value determines the system's sensitivity to human mobility. Figure 12 shows the localization errors at different *frameTh* values. The *x*-axis is the *frameTh* values and *y*-axis is localization error. When *frameTh* increases, we observe a decrease in localization errors for Sensors 1 and 2 (Sensor 1: 0.38, 0.38, 0.35, 0.35, and 0.38 m; Sensor 2: 0.36, 0.31, 0.26, 0.25, and 0.21 m). The impact on Sensors 3 and 4 is not clear, this is because the events selected by Sensors 3 and 4 (high SNR vibration signals) will be farther from the camera than those selected by Sensors 1 and 2. When footsteps are farther away from the camera, more motion will lead to less change in pixels, which in turn will reduce *motionTh*'s impact on footsteps selection. When *frameTh* increases to 19, the localization error for Sensors 3 and 4 decreased to 0.35 and 0.40 meters respectively. This could be because only very stable footsteps are detected; and to a certain extent, this filters out high fidelity data and hence improves the accuracy. Figure 13 shows the localization success rates are all greater than 0.9 for the investigated *frameTh* values, which demonstrates our system's robustness. To further understand how this parameter impacts system performance, we plot the number of traces being sampled in order to extract at least four footfall events that meet the signal fidelity constraints in Figure 14. We observe that the number of traces sampled, increases with the *frameTh*. For Sensors 1 and 2, the average number of traces needed to successfully localize the sensors increases from 5.7 to 13 as *frameTh* increase from 11 to 19 frames. Sensors 3 and 4 demonstrate the same trend, but show a smaller increment in the number of traces (6.21 to 7.26 traces). This increasing trend is because when *frameTh* increases, the number of detected footfall events decrease, but the number of true positive events increases. As a result, more traces need to be sampled in order to acquire enough events for sensor localization. *AutoLoc* takes *frameTh* = 15 as the parameter setting taking into account both the localization error and data usage efficiency.

#### 4.3.3. Impact of Numbers of Refine Iteration

**Figure 15:**
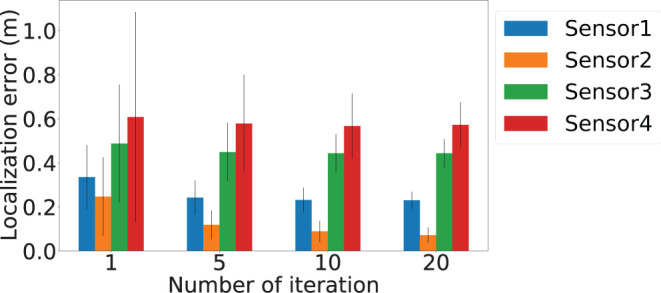
Number of iteration and its impact on localization errors. The *x*-axis is the number of iterations of estimation used for sensor localization, and *y*-axis is the localization error. Blue, orange, green, and red bars represent results of Sensor 1, Sensor 2, Sensor 3, and Sensor 4, respectively.

**Figure 16:**
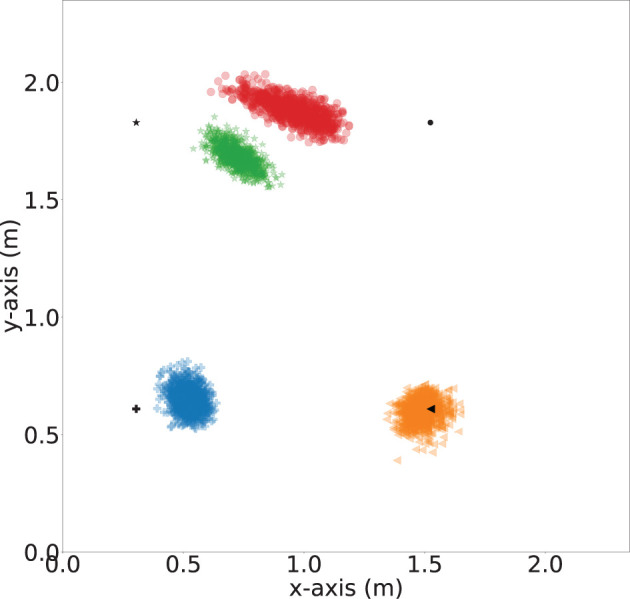
Sensor localization visualization (with 20 iteration refinement). Blue, orange, green and red dots represent estimations of Sensor 1, Sensor 2, Sensor 3, and Sensor 4, respectively. Black dots represent ground truth locations.

To demonstrate the importance of the iterative location update module, we further evaluate the impact of number of refine iteration *k*. In order to understand the impact of the iterative refinement of the location estimation, we depict the localization error values after different numbers of iterations in Figure 15. Bars in four different colors are used to represent the average of the localization error and the error bars represent the standard deviation of the errors of the four sensors. We observed a general decreasing trend across all the sensors when the number of iteration increased. The highest improvement is for Sensor 2, where the average localization error reduced by 0.175 meters when the number of iteration increases from *k* = 1 to *k* = 20, which is an 2.5× improvement. Besides, the standard deviation decrease from 0.17 to 0.03 meters, which indicates a more stable estimation. To intuitively demonstrate the localization performance, we plot the successfully localized sensor location with 20 iterations of refinement in Figure 16, where black dots represents the ground truth. Blue, orange, green, and red dots depict refined sensor location estimation.

## 5. Discussion

We discuss the limitation and future direction of *AutoLoc*.

### 5.1. Simultaneously Synchronization and Localization

In this work, we assume the devices are synchronized, which may not be true for many heterogeneous systems. When the synchronization resolution between sensors are low, the TDoA estimation between pairwise sensors may be impacted and reduce the sensor localization accuracy. We plan to explore reinforcement learning approach in the future to tackle this problem, where the system takes footstep localization *via* given sensor localization results as the reward and selection of the clock shift values as actions. In this way, the clock shift can be estimated over iteration and the clocks synchronization is improved.

### 5.2. Occlusion-Aware Footstep Event Detection

In this work, we do not assume the line-of-sight (LoS) between the camera and the vibration sensors, but we do assume the LoS between the camera and the occupant. However, when deployed at home scenario, especially when there is furniture around, this assumption may no longer hold. When partial occlusion occur, the posture estimation module may fail to output the posture keypoints, or output erroneous keypoint locations. This may impact our system's performance, since *AutoLoc* relies on the detected heel keypoints for footstep event detection. Recent work on posture estimation addresses the explicit occlusion issue estimation with explicit occlusion *via* occlusion-aware posture estimation (Cheng et al., 2020; Zhang et al., 2020), which can be applied in our system to enhance the system robustness. In addition, since the occupants move around, there are areas or frames with opportunistic LoS between the camera and occupant, which can be used for sensor localization when the events accumulate.

### 5.3. Automatic Sensing Area Detection

In this work, we manually select the sensing area on the floor in the camera view. However, this procedure can be potentially automated with prior work of the ground detection (David, 2008; Wang et al., 2016) and vanishing point analysis (Gerogiannis et al., 2012). Recent work on indoor layout estimation can also provide powerful detection tools for this purpose with the assistance of the LiDAR sensor (Li and Stevenson, 2020). We envision that with various sensors installed—both direct (e.g., camera, LiDAR) and indirect (e.g., vibration, mmWave, magnetic) sensing approaches—the inference of the environment and people in it can form a holistic view of the physical world. Built upon that, the IoT systems can further achieve autonomous configuration with zero human interaction.

In this work, we do not focus on the environment (e.g., room layout) modeling part of the IoT system automation. Instead, we focus on the association of information across different modalities and enable auto configuration of “indirect” sensing modality—floor vibration based human sensing. In the future, we plan to explore how multiple sensing modalities can further achieve each others' optimum configuration and information inference accuracy.

## 6. Conclusion

In this paper, we present *AutoLoc*, a cross-modal vibration sensor location configuration scheme. *AutoLoc* utilizes the ambient occupant sensing information—footstep locations—captured by co-located cameras as the shared context to achieve autonomous vibration sensor location estimation. A spatial characterization-based data selection scheme is applied to further enhance the location estimation accuracy. Real-world experiments are conducted to evaluate *AutoLoc*. *AutoLoc* achieves an up to 0.07 meters sensor localization accuracy and demonstrates an up to 4× improvement compared to baselines.

## Data Availability Statement

The raw data supporting the conclusions of this article will be made available by the authors, without undue reservation.

## Ethics Statement

The studies involving human participants were reviewed and approved by Office of Research Compliance and Integrity, University of California, Merced. The patients/participants provided their written informed consent to participate in this study.

## Author Contributions

SR, YZ, CR, and SP: conception and design of the study. SR, YZ, and SP: drafting and revising the manuscript. SR and YZ: acquisition of data. SR: analysis and/or interpretation of data. All authors contributed to the article and approved the submitted version.

## Conflict of Interest

CR was employed by Aifi Inc. The remaining authors declare that the research was conducted in the absence of any commercial or financial relationships that could be construed as a potential conflict of interest.

## Publisher's Note

All claims expressed in this article are solely those of the authors and do not necessarily represent those of their affiliated organizations, or those of the publisher, the editors and the reviewers. Any product that may be evaluated in this article, or claim that may be made by its manufacturer, is not guaranteed or endorsed by the publisher.
